# Glioblastoma, from disease understanding towards optimal cell-based *in* *vitro* models

**DOI:** 10.1007/s13402-022-00684-7

**Published:** 2022-06-28

**Authors:** Chiara Boccellato, Markus Rehm

**Affiliations:** 1 Institute of Cell Biology and Immunology, University of Stuttgart, Allmandring 31, 70569 Stuttgart, Germany; 2 Stuttgart Research Center Systems Biology, University of Stuttgart, 70569 Stuttgart, Germany

**Keywords:** Glioblastoma, Cancer, *Invitro* models, Cell systems

## Abstract

**Background:**

Glioblastoma (GBM) patients are notoriously difficult to treat and ultimately all succumb to disease. This unfortunate scenario motivates research into better characterizing and understanding this disease, and into developing novel research tools by which potential novel therapeutics and treatment options initially can be evaluated pre-clinically. Here, we provide a concise overview of glioblastoma epidemiology, disease classification, the challenges faced in the treatment of glioblastoma and current novel treatment strategies. From this, we lead into a description and assessment of advanced cell-based models that aim to narrow the gap between pre-clinical and clinical studies. Such *in* *vitro* models are required to deliver reliable and meaningful data for the development and pre-validation of novel therapeutics and treatments.

**Conclusions:**

The toolbox for GBM cell-based models has expanded substantially, with the possibility of 3D printing tumour tissues and thereby replicating *in* *vivo* tissue architectures now looming on the horizon. A comparison of experimental cell-based model systems and techniques highlights advantages and drawbacks of the various tools available, based on which cell-based models and experimental approaches best suited to address a diversity of research questions in the glioblastoma research field can be selected.

## Glioblastoma (GBM) epidemiology and classification

Glioblastoma is the most common form of primary malignancy of the central nervous system (CNS) in adults. It was described in 1863 by the German pathologist Rudolf Virchow [1], who identified it as is a form of glioma. Gliomas are tumours arising from glial or precursor cells and comprise astrocytomas—with glioblastoma being one of them -, oligodendrogliomas and ependymomas. Although considered a rare tumour, with an incidence rate of less than 5 cases per 100,000 people, glioblastoma accounts for 14.5% of all brain and CNS tumours and for 48.6% of the malignant ones. According to recent statistics of the central brain tumour registry of the United States (CBTRUS; data for 2014–2018) it affects men more than women, the median age at diagnosis being 65 and the five-years survival rate being of approximately 6.8% depending on patients’ characteristics and tumour histology [2].

Glioblastomas are mostly found in the cerebral hemispheres, especially in the frontal and temporal lobes, while only a few percent occur in the cerebellum, brainstem and the spinal cord [3]. Its infiltrative properties are long known, and difficulties in identifying a discrete border zone between the tumour and the normal brain parenchyma were already reported in 1928 [4]. Depending on the functional role of the area of the brain affected, clinical presentations include persistent weakness, numbness, loss of vision or alteration of the language. Headache is a very common initial symptom while seizure only occurs in approximately 25% of patients [5].

The aetiology of this tumour type is still obscure. Only 20% of glioblastoma patients have a family history of cancer and the definition of susceptibility or predisposition genes remains challenging [6]. However, some familial cases have been found to be associated with rare genetic syndromes like Li-Fraumeni syndrome and neurofibromatosis of types 1 and 2 [7]. Among the environmental and dietary risk factors assessed, only high doses of ionizing radiation have been confirmed as such, while no association could be found for lifestyles that include alcohol or drug use, cigarettes smoking or specific diets [8, 9].

The term glioblastoma was used for the first time in 1927 by the neuropathologist Percival Bailey and the neurosurgeon Harvey Cushing, who provided the first systematic classification and histological description of gliomas [10]. Since then, several updates on more appropriate classification and nomenclature systems followed. Most recently, in 2021, the world health organisation (WHO) revised the classification of CNS tumours (WHO CNS5) by including molecular parameters in addition to histological features for the definition of diagnostic categories and a grading system [11]. Of note, the nomenclature was already revised in the fourth edition of the WHO Classification of Tumours of the Central Nervous System and the term “multiforme” was abolished, even though the abbreviation “GBM” is still widely used [12]. Based on the WHO CNS5 classification, a mutated IDH gene now separates astrocytomas (specifically astrocytoma, IDH-mutant of CNS WHO grade 4) from glioblastomas. IDH1 is a protein found in the cytoplasm, peroxisomes and endoplasmic reticulum, where it catalyses the oxidative decarboxylation of isocitrate to α-ketoglutarate. IDH2 has a function similar to IDH1, but is found in mitochondria. In contrast to IDH mutated astrocytoma, glioblastomas are defined as an adult-type of diffuse astrocytic tumours displaying a wild-type status of the IDH gene (Glioblastoma, IDH-wildtype) and assigned to CNS WHO grade 4. More precisely, in the setting of an IDH-wildtype diffuse and astrocytic glioma in adults, a glioblastoma is diagnosed if microvascular proliferation or necrosis, TERT promoter mutation, EGFR gene amplification or a combined gain of the entire chromosome 7 and loss of the entire chromosome 10 [+ 7/ − 10] is observed.

## The challenges of GBM treatment and classification

The treatment of glioblastoma is extremely challenging. The difficulties in finding an effective therapy are mainly due to four reasons: its invasive properties, its heterogeneity, its rapid development of resistance to radio-chemotherapy, and the presence of the blood–brain barrier (BBB), which most drugs cannot cross.

Glioblastoma cells have the ability to infiltrate into normal brain tissue, penetrating the brain parenchyma and the perivascular space by degrading the extra cellular matrix (ECM). Due to their aggressive migrative behaviour, glioblastoma cells escape complete surgical resection and, therefore, the tumour usually re-occurs within a few centimetres from its original location [13, 14]. Numerous studies have been conducted on glioblastoma invasiveness, trying to clarify patterns and directionalities as well as the mechanisms responsible for the invasive behaviour. For example, it was found that while certain brain regions are more frequently invaded by glioblastoma cells, others such as the hippocampus are normally spared [15]. The processes that allow glioblastoma cells to invade surrounding tissues have been extensively studied and are reviewed elsewhere [16]. In general, they comprise cell-to-cell and cell-to-ECM adhesion mechanisms, ECM and cytoskeletal remodelling and overall features of epithelial-mesenchymal transition (EMT). Unfortunately, possibly due to the complex networks of the signalling pathways involved, this knowledge could not yet be translated into efficient therapeutic strategies in the clinic.

Tumour heterogeneity encompasses both inter-tumour heterogeneity, which refers to the distinct genetic alterations and differing tumour architectures found among different patients, and intra-tumour heterogeneity, which refers to the diversity of cell-to-cell and tissue contexture within an individual tumour. Genome wide transcriptome analyses of high-grade gliomas has led to the classification of three subtypes that could be prognostically relevant [17–19]. These are referred to as the proneural (PN), classic (Class) and mesenchymal (Mes) subtypes. An additional subtype, the so-called neural (Neu) subtype, has been removed from the classification as it could be traced by normal neural lineage contamination [20–22]. Treatment outcomes among individuals of the same tumour subtype are not homogeneous, likely due to the abovementioned heterogeneities. One caveat of the subtyping process is that classification must be based on the analysis of a specific site of the tumour from which a biopsy is taken. However, biopsies from different regions of the tumour of a patient can result in the assignment to a different molecular subtype [23, 24]. Moreover, single-cell RNA analysis has shown that tumours can comprise cells of all three subtypes [25, 26]. Recent spatial analyses of tumour heterogeneity revealed that the peripheral (vascular) portion of the tumour core preferentially expresses proneural genes while the central core portion (hypoxic region) more frequently consists of cells that can be assigned to the mesenchymal subtype [27]. A more detailed description of the features of distinct regions of a glioblastoma tumour can be found in the anatomical atlas that was compiled in 2018 by Puchalski and colleagues, who integrated mutation and gene expression data obtained from morphologically distinct parts of the tumour [28]. Due to the pronounced intra-tumour heterogeneity, it is understandable that among the different sub-populations that coexist within a tumour some are spared from the treatment and can cause relapse. Hence, this adds another level of complexity to prognostication, but at the same time exemplifies why interest in and understanding of intra-tumour heterogeneity continues to increase [29]. In an effort to categorize this complexity, interesting work has been conducted by Bergmann and colleagues [30]. Based on the immunoreactivity to nine relevant markers (AlDH1; CA-IX; EGFR; GFAP; MAP2; Mib1; Nestin; NeuN; Vimentin), they found that different areas of a glioblastoma tumour could be clustered into five pathophysiological groups that reoccur throughout the tumour mass. Interestingly, the characteristic marker profile of these groups can be aligned with that of the abovementioned glioblastoma subtypes (PN; Class and Mes), thereby offering a mean to bridge the divide between intra-tumour and inter-tumour heterogeneity classifications. Importantly, as the five groups do not exclude each other but rather co-exist in diverse regions of the same tumour, it needs to be noted that glioblastoma subtyping without spatial contexture can inappropriately homogenize the intra-tumour heterogeneity and will be dominated by tumour regions that are quantitatively predominant in the respective biopsies.

A common assumption was that the source of tumour heterogeneity could be ascribed to a population of cells referred to as cancer stem cells (CSCs), which thus became targets for the development of therapeutic strategies [31]. Glioma stem-like cells (GSCs) own their name to the similarities with normal neural stem cells (NSCs) in terms of self-renewal properties, ability of differentiate into different cell types, expression of neurogenic markers like CD133, CD15 and Nestin and transcriptional stemness factors including Sox2, Olig2, Nanog and c-Myc [32–37]. By employing single-cell transcriptomics, Bhaduri and colleagues [38] showed the existence of heterogeneous GSCs subtypes that co-exist within a single tumour and, among them, they identified the outer radial glia (oRG) cells as an invasive population with a behaviour that is typically only observed during human development. Together with the presumed reactivation of developmental programs in these cells, they concluded that heterogeneous GSCs may be responsible for the aggressive invasive properties of glioblastoma. However, as reviewed by Rich [39], both the definition of cancer stem cells itself and also how they give rise to tumour heterogeneity are still matters of debate. The emergence of the concept of cellular plasticity implies a dynamic equilibrium between GSCs and differentiated non-GSCs, with a potential for non-GSCs to revert (dedifferentiate) back into to GSCs [40–42]. For example, it has been reported that conditions like hypoxia- or radiation-induced stress can promote dedifferentiation into a stem-like cell state [43–45]. That is why recent studies warn against the exclusive targeting of stem-like cell subpopulations, as these need to be considered “moving targets” with a state that adapts to changing microenvironments [46]. Instead, efforts are underway to better understand the plasticity of tumour cells and the mechanisms that influence their state transitions [47]. The process of these transitions, rather than aiming at the so-called stem cells, might indeed be promising targets for designing improved treatments to prevent tumour re-occurrence and repopulation.

Another feature that complicates the treatment of glioblastoma is the presence of the BBB. The BBB is a blood-to-brain interface that mechanically and biochemically segregates the brain from the parenchyma, strictly controlling the passage of substances in and out of the CNS. This physical separation is achieved by special brain capillary endothelial cells which do not have fenestrae and form a tight barrier that most polar, water-soluble molecules bigger than 450 Da cannot cross [48]. As such, the BBB also protects the CNS from harmful compounds or infections. Of note, the BBB endothelial cells express proteins of the ATP-binding cassette (ABC) superfamily that hydrolyse ATP to actively pump out substances against their concentration gradient. These integral membrane transporters can recognise a wide variety of substrates and, thanks to this low specificity, they carry out a crucial function in protecting the brain from diverse toxins. Unfortunately, since this can also impair the passage of therapeutics, ABC transporters can contribute to the establishment of drug resistance [49–51]. In fact, ABC transporters at the BBB, but also in tumour cells, are often highly expressed and enhance the multidrug resistance of glioblastoma [52–54]. For example, P-glycoproteins (P-gp), that are encoded by the ABCB1 (or MDR1) gene, typically recognise hydrophobic substrates, including traditional drugs used for the treatment of glioblastoma such as the alkylating agent carmustine and the standard of care temozolomide (TMZ) [55, 56]. Many chemotherapeutics are also recognized by the breast-cancer resistance protein (BCRP), which is encoded by the ABCG2 gene. Interestingly, BCRP shares some substrate specificity with P-gp and with the multidrug resistance protein 1 (MRP1/ABCC1), a redundancy that has probably evolved to ensure protection also in case of mutations in any of these proteins [57, 58]. In summary, these transporters play a crucial role in preventing therapeutics from reaching the brain and the target sites of the tumour.

## GBM treatment options and novel treatment strategies

GBM patients are notoriously difficult to treat and progress towards novel and improved treatment options is slow. Currently, the standard of care is still based on the Stupp protocol which more than fifteen years ago proved that the addition of concomitant and adjuvant TMZ to fractionated focal irradiation of 60 Gy improves overall survival from 12.1 to 14.6 months compared to radiotherapy alone [59]. TMZ exerts its cytotoxicity by transporting a methyl group that attaches to guanines at their O^6^ position during the process of DNA replication. By this, an O^6^-methylguanine (O^6^-MG) is formed which then pairs with a thymine base nucleotide instead of a cytosine. Such a mismatched base pairing provokes DNA breaks, with consequent cell cycle arrest at the G_2_/M transition of the cell cycle, followed by apoptotic cell death. Radio-chemotherapy with TMZ, followed by six cycles of adjuvant TMZ and preceded by maximal safe surgery, is nowadays still the first line treatment for glioblastoma. Unfortunately, the survival benefit provided by TMZ is very modest and many patients do not respond at all to such treatment. Innate or acquired resistance to TMZ has been investigated comprehensively [60]. A follow-up report on the Stupp protocol study and also the more recent DIRECTOR trial on recurrent glioblastoma identified the methylation status of the promoter of the O^6^-methylguanine-DNA methyltransferase (MGMT) gene as a positive prognostic marker of TMZ responsiveness [61, 62]. MGMT is a DNA repair enzyme that removes alkyl and methyl adducts formed at the O^6^ position of guanines, thereby antagonising the lethal effects of alkylating agents like TMZ. MGMT protein expression is under epigenetic control, meaning that the methylation of its promoter results in the silencing of the gene. As it was found that MGMT promoter methylation correlates with improved progression-free and overall survival in patients treated with alkylating agents, several studies have focused on trying to modulate the expression of this enzyme [63, 64]. Interestingly, the methylation status of MGMT changes throughout the progression of a tumour, and can be affected by TMZ treatment itself [65, 66]. In particular, it has been shown that tumours with an initial methylation of the MGMT promoter frequently reoccur with a decreased methylation if treated with TMZ [67, 68].

The resistance to TMZ, together with the practical infeasibility of surgically removing the entire tumour mass, inevitably leads to relapse. Although the treatment of recurrent glioblastomas is not standardised, a follow up surgery (when feasible) and/or radiotherapy (rare) can be indicated in selected patients. The second line chemotherapy consists of either nitrosourea-based regimens, like lomustine (CCNU) alone, or the combination of lomustine plus alkylating agents (like TMZ) plus bevacizumab, or bevacizumab alone [69–72]. The use of lomustine in the treatment of recurrent glioblastoma provides a median overall survival of 8 to 9 months and a median progression-free survival of almost 3 months [72, 73].

Bevacizumab (brand name Avastin®) is a recombinant humanized anti-vascular endothelial growth factor (VEGF) monoclonal antibody. By binding to circulating VEGFs it inhibits their recognition by the respective cell surface receptors, thereby reducing the growth of blood vessels [74, 75]. Bevacizumab was first studied for the treatment of metastatic colorectal cancer, and in 2006 received approval of the American food and drug administration (FDA) [76]. Later, its use against glioblastoma was evaluated in several clinical trials, both for recurrent cases and as a first line treatment, but with no reported improvement in the overall survival of patients [77, 78]. It was hypothesized that the limited efficacy of bevacizumab, as well as that of other monoclonal antibodies like the EGFR inhibitor cetuximab, might be due to their size and, therefore, difficulty in penetrating the BBB [79].

Several studies have focused on common mutations in glioblastoma and the possibility to devise associated treatment regimens. Tyrosine kinase receptor pathways are also among the most frequently mutated pathways in glioblastoma and strategies to target them with the small multi-kinase inhibitor Regorafenib are now being evaluated in the AGILE and Regoma (NCT03970447 and NCT02926222) studies, with promising initial results [80]. In the human genome, genes for three neurotrophic tyrosine receptor kinases (NTRK) can be found, and in cancer cells these can appear as fusion genes that lead to constitutively active TRKs that drive tumour cell proliferation [81]. NTRK inhibitors targeting NTRK fusion-positive cancers are currently under study in glioblastoma [82].

Glioblastoma displays an overall strong resistance to conventional therapies. Hence, additional strategies to eradicate this tumour are under development. One of the most intensely studied therapeutic approaches are immunotherapeutic treatments [83]. In GBM, these include trials with immune checkpoint inhibitors that initially were approved for the treatment of metastatic melanoma, but also efforts to develop vaccines based on tumour antigens, as has been reviewed extensively elsewhere [83, 84]. Unfortunately, results so far have been disappointing, probably owing to the low overall mutation burden of GBM and its immunosuppressive microenvironment. Also limited T cell infiltration and T cell exhaustion have been brought forward as factors limiting the success of established immune-therapeutics in glioblastoma. Attempts to develop CAR T cell therapies, in which patient T cells are genetically modified and trained to recognize patient-specific tumour antigens, likewise are being studied, with limited success in subsets of patients [84]. Oncolytic virotherapy is another approach that prospectively could provide advances to glioblastoma disease management and therapy [85]. Oncolytic viruses (OVs) can destroy the tumour cells in which they are hosted (oncolysis), and the consequent release of tumour antigens stimulates the immune system. The possibility of modifying OV genomes allows arming them with cell suicide genes [86]. Since the first virus for oncolytic purposes was engineered in 1991, oncolytic virotherapy has developed substantially [87]. Apart from Herpes simplex, many other viruses have been trialled and this led, in 2015, to the first FDA-approved oncolytic virus for the treatment of melanoma [88, 89]. In glioblastoma, viruses from ten different families have been tested, some with very promising results [90]. For example, PVS-RIPO is an engineered variant of the poliovirus type 1 vaccine whose internal ribosomal entry site (IRES) has been replaced with that of the human rhinovirus type 2 in order to reduce its neurotoxicity. The entry site of the virus is the cell receptor CD155/NecI5 which, by being upregulated in glioblastoma cells, provides the necessary tumour specificity and safety profile that allows the use of a poliovirus as a therapeutic agent [91, 92]. This recombinant non-pathogenic polio-rhinovirus chimera proved to be very effective in early stages of clinical trials, with 20% of patients still being alive three years after initiating the treatment [93].

Among the approved treatments against glioblastoma, it is also relevant to mention the Tumour Treating Fields (TTFields). This treatment modality consists of the delivery of low intensity (< 3 V/cm) and medium frequency (200 kHz) electric fields that alter spindle formation and, subsequently, lead to mitotic arrest or cell division delay [94]. The first FDA-approved TTF device was the NovoTTF-100A (Optune®), pioneered by the company Novocure. Clinical studies have shown that the addition of TTF to TMZ increased patients’ overall survival compared to chemotherapy alone, paving the way for more studies on this technology [95–97].

As mentioned above, the difficulties in treating glioblastoma are also due to the presence of the BBB, a physical and cell biological obstacle to the delivery of drugs to the brain. Upon brain metastasis or primary brain tumour development, the brain environment undergoes changes affecting features of the BBB, such as its structure and functionality. As these alterations are guided by cancer cell behaviour, in these circumstances the blood-to-brain barrier is referred to as the blood-to-tumour barrier (BTB). In contrast to the traditional understanding of the BTB simply as a non-orchestrated breakdown of the BBB, evidence of consistent alterations in permeability and composition is now emerging [98]. This includes for the example the downregulation of various efflux transporters, which possibly could be therapeutically targeted in the future to improve drug penetration [99–101]. However, as of now the BTB has only been poorly characterised, and strategies for the delivery of drugs into the brain focus on BBB permeable drugs, exploiting physiological trans-BBB transport processes such as transcytosis or on breaking down the BBB or BTB impermeability. For example, considering that the first line treatment of basically every glioblastoma patient involves surgery, implantable polymers can be placed in the tumour cavity at this stage. Implants loaded with the chemotherapeutic carmustine have been developed and are available as Gliadel® Wafers. These wafers have been clinically tested, also in combination with TMZ, and already received FDA approval [102–104]. Nevertheless, modalities to improve drug delivery to the brain represent a growing area of research, with hydrogel and nanocarrier technologies being optimised in the last years for their use against glioblastoma [105].

Finally, it is important to mention that non-invasive ways of temporarily opening the BBB to allow drug penetrance are also under investigation. In particular, the BBB can be disrupted in a controlled manner by using focused ultrasounds. The safety of this technology has started to be assessed in both mice and humans and ultrasound delivering devices, like the SonoCloud by CarThera, are now under clinical evaluation (NCT04614493) [106, 107].

The above list of therapeutic options is not exhaustive, but serves to highlight the scarcity of approved therapies for glioblastoma and, thus, the urgent need to find new effective therapies.

## *In vitro* GBM models for experimental research

**Fig. 1:**
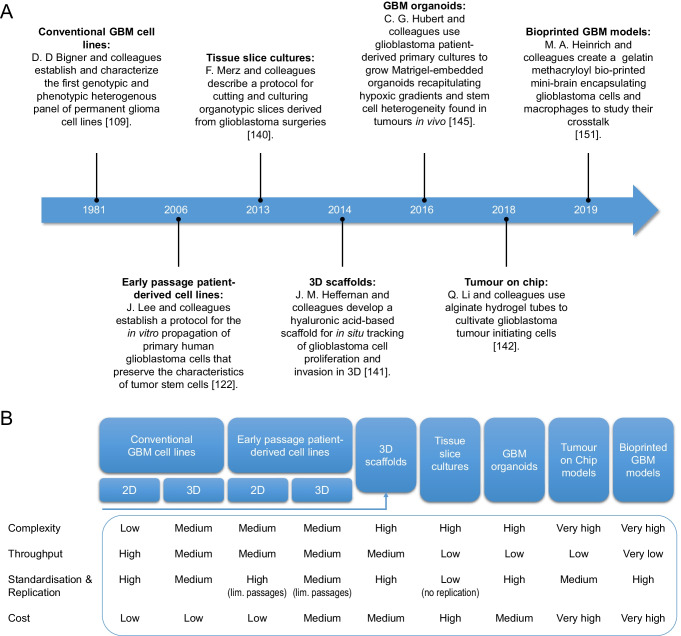
Overview of GBM cell-based *in* *vitro* and *ex* *vivo* models. (**A**) Time line highlighting key mile stones in the development of cell-based GBM models. (**B**) Scheme providing an overview of common and advanced cell-based *in* *vitro* and *ex* *vivo* models. The respective model systems were assessed for complexity, suitability for high throughput analyses, standardisation and replication, as well as overall costs

One of the reasons for the limited success of novel drug candidates entering clinical trials is the poor correlation between their efficacy in *in* *vitro*, *ex* *vivo* or *in* *vivo* tumour models and patient treatment scenarios. Cell culture methods often fail to capture all aspects that contribute to the complexity of glioblastoma. Hence, depending on the question to be addressed experimentally, the most suitable model system will need to be selected. Concerning pre-clinical *in* *vitro* models, multiple options are available (Fig. 1A). These span across different cell- and cell line-based modes and culture conditions. Below, we provide an overview of experimental *in* *vitro* models that are available for glioblastoma research.

### GBM cell line models

Traditionally, cell-based studies on glioblastoma have been performed on immortalised cell lines, most commonly U-87 MG, U-251 MG, LN-229 or A172 [108]. Such widely used cell lines have been developed from patient tumours often decades ago, sometimes also by artificially manipulating the cells such that they would proliferate indefinitely. The advantage of these well-characterized cell lines is that they constitute a simple system comprising one single cell type that can be maintained easily in serum-containing medium. This supports experimental replicability and reproducibility, and also comparative studies between multiple international laboratories. Commercially available cell lines are a continuous source of cell material, therefore allowing large scale studies such as drug screens. Moreover, the content of so-called GSCs can be increased in such cells lines, for example when culturing these as spheroids, thereby allowing studies on stem-like subpopulations [115]. Unfortunately, established cell lines come with drawbacks. First, as they have been established a long time ago, back tracing and authentication of such cell lines sometimes is difficult. Genetic analyses of the cell line U-87 MG revealed, for example, that although it is considered to be a *bona* *fide* human glioblastoma cell line, its actual origin is uncertain [116]. While established in 1966 at Uppsala University in Sweden from what was thought to be the glioblastoma of a 44-year-old woman, fifty years later it is available in the American Type Culture Collection (ATCC) as an authenticated cell line derived from a male patient [117]. Another important limitation is that such cell lines have been passaged *in* *vitro* uncountable times. As such, they have been selected over time for those with the highest proliferation rate, and at the expense of the genetic heterogeneity that is a hallmark of glioblastoma and that might have been present in the original isolates. Moreover, successive and prolonged passaging can lead to genetic drift, which may result in alterations in both the genotype and phenotype of these cells, thereby broadening the gap between the cell line model and the actual *in* *vivo* tumour [118, 119]. Likewise, the commonly used serum-containing medium provides an environment to which glioblastoma cells otherwise would not be exposed and which can substantially alter their transcriptome and proteome [120, 121]. Nevertheless, established glioblastoma cell lines can be considered as convenient models for a fast screening of drug candidates and for obtaining preliminary results. Clearly, however, these would need to be validated further in more advanced experimental GBM models.

### Early passage patient-derived cells

The use of primary early passage cells obtained from fresh tumour samples is becoming more common and begins to replace the use of traditional cell lines. Such cell materials are often referred to as patient-derived cell lines (PDCLs), even though obviously also traditional cell lines are derived from patient materials. To obtain PDCLs, fresh biopsies are processed within 2–3 h post-resection based on protocols that preserve the characteristics of the original tumour [122, 123]. PDCLs are now considered state-of-the-art preclinical models for glioblastoma studies, as they have been found to maintain the gene expression profiles present in the parental tumour [124, 125]. Nevertheless, depending on the conditions adopted for their subsequent culturing, the behaviour of these cells and the fraction of GSCs within the cultures can vary broadly [126]. One major issue arises from the use of serum-supplemented media, which reduces the relative proportion of GSC subpopulations and appears to induce their differentiation into more committed cells [112, 127]. This can be avoided by culturing PDCLs in the absence of serum and, instead, in the presence of defined supplements. The most commonly used medium for this purpose is Dulbecco's Modified Eagle Medium (DMEM) in 1:1 ratio with Nutrient Mixture F-12. The growth factors typically added to this medium comprise epidermal growth factor (EGF) and basic fibroblast growth factor (bFGF), plus additional supplement such as N2 or B27, which contain lipid components that promote the proliferation of glioblastoma cells [128]. Compared to standard cell cultures, the maintenance of cells in serum-free, supplemented medium, has been shown to preserve the genetic aberrations and gene expression profiles of the tumours of origin [112, 124]. For a PDCL to be considered a reliable model of a glioblastoma tumour, its culture time should be limited to a maximum of about twenty passages, since higher passages may alter their transcriptional profiles [129].

Glioblastoma PDCLs can be cultured either as monolayers (2D) or as spheroids (3D). 2D cultures allow an efficient propagation of cells that are homogeneously exposed to the medium. Cells in 2D cultures exhibit a different morphology compared to those grown as 3D models and, therefore, drug sensitivities may differ between these conditions [130]. However, this might not always be the case, so the requirement for 3D-cultured PDCLs as models for the screening of drug responsiveness in glioblastoma needs to be assessed on a case-by-case basis [131, 132].

The culture of PDCLs in 2D can be supported by treating the plastic of the culture flasks with coating polymers that reduce the stiffness of the surfaces. This can be of relevance when studying invasion and proliferation, since the unnaturally stiff environment can alter these processes [133, 134]. To provide an environment that more closely resembles that of the tumour, hydrogels containing different ECM components have been developed. For example, Matrigel is a natural hydrogel derived from the ECM of mouse sarcoma tumours and has been adapted for culturing glioblastoma cells. Matrigel, just like other ECM-derived hydrogels that have been developed, contains mostly laminin and collagen IV [135]. This composition is well-suited to mimic the ECM of several tissues, but does not appropriately reflect that of the brain. Indeed, the brain ECM is richer in proteoglycans and glycoproteins, especially hyaluronic acid and heparan sulfate. On the other side, laminin is the main component of the wall of the blood vessels and GSCs are known to mostly reside in perivascular niches [136]. Therefore, the use of such coating polymers still provides a valid approximation of the brain environment and is preferable over plastic surfaces.

Overall, the short-term culture of PDCLs in EGF and bFGF-supplemented serum-free medium and on ECM coatings represents the best and cheapest option to recapitulate the characteristics of the parental tumour. Moreover, PDCLs allow for the analysis of tumour cell diversity among individual patients, which is a step closer towards the study of inter-tumour heterogeneity. Considering that PDCLs can also grow as spheroids, 2D PDCL models can be complemented with 3D culture systems.

### 3D tumour cell models

2D cell culture models have the intrinsic drawback of oversimplifying the complex cell-to-cell interactions found in tumours [137, 138]. In order to circumvent this problem, serum-free cultured glioblastoma PDCLs can be grown suspended in medium over non-adhesive substrates, such as ultra-low attachment plates or culture ware treated with anti-adherence rinsing solutions. GBM cells then form typical aggregates referred to as spheroids, or more specifically neurospheres (NS) or gliomaspheres. Spheroids kept under these conditions have been shown to maintain cellular subpopulations with stem-like properties [139]. Spheroids originate from the spontaneous aggregation of multiple single cells and can vary greatly in size and thus cell number, an aspect to be taken into account when analysing the differences or similarities in cellular responses between spheroids and between repeat experiments [140]. If clonal identity between spheroid cells is required, limited dilution assays can be employed [141]. These allow to grow spheres from individual starting cells and also provide an indication for the tumour initiating capacity of such cells [142]. In a simplified view, cells that depend on cell-to-cell contacts for continued growth would be assumed to undergo anoikis, whereas GSCs would be expected to grow in suspension in serum-free medium and to form spheres. However, in conditions of low cell density paracrine signals are missing, cell growth can be very slow and can come at the cost of losing cellular heterogeneity of the starting cultures or cell isolates. Still, PDCLs grown as spheres/spheroids are a relatively cheap and convenient model that, especially when used in combination with 2D PDCLs, represent a widely used standard employed in many laboratories for pre-clinical studies.

### GBM slice culture models and 3D scaffolds

More elaborated experimental models have been developed to overcome the limitations of neurospheres. For example, organotypic glioma slice cultures directly derived from biopsies comprise several cell types and structures of the glioblastoma microenvironment like non-malignant cells, ECM and blood vessel structures. Because of these features, organotypic slice cultures are particularly suitable for studying migration and invasion properties of glioblastoma cells within a more natural microenvironment that remains genetically stable [109, 143]. On the downside, slice cultures are cost and time consuming, individually unique and due to their nature as tissue samples do not lend themselves for repeat experiments or for well-controlled reference experimental systems.

In order to study cell-to-cell as well as cell-to-matrix interactions in a 3D model, various hydrogel-coated scaffolds have been developed with different biomaterials and coatings that try to mirror the brain ECM. The list ranges from matrigel-coated polystyrene scaffolds to hyaluronic acid ones. Relatively cheap and easy to scale up for high-throughput experiments, these scaffolds already found several applications, such as drug response studies and proliferation and invasion experiments [113, 130]. However, the stiffness of the scaffold, that is known to influence cell behaviour, represents a limitation of these models. Additionally, throughout cell passaging, an inevitable selection process takes place that favours those cells that attach more loosely to the matrix and that, therefore, are more easily retrieved, an effect that may bias invasiveness studies.

### Tumour-on-chip models and GBM organoids

A tumour is a dynamic entity that adapts to changes in the microenvironment. 3D cultures instead, are far more static because the surrounding medium conditions remain largely stable. To represent the variability of the tumour microenvironment, and therefore the variable conditions to which tumour cells are exposed, microfluidic systems like “tumour-on-chip” models have been developed. In such systems, cells are grown in hydrogel tubes filled with circulating media whose composition can be time-controlled in terms of nutrients and factors to be added. In addition, brain-specific ECM components, like hyaluronic acid, can be included to create a more realistic glioblastoma microenvironment. This technology has been applied for the long-term cultivation (> 50 days) of glioblastoma tumour initiating cells (TICs), such that stem cell properties could be maintained. Cells can be pumped into alginate hydrogel tubes (AlgTubes) and continuously grown to form strands of spheres expressing GSC markers [114]. Hence, this may represent an efficient method for the mass production of TICs.

A recently emerged platform for the study of glioblastoma development and its pathology is represented by brain organoids or “mini-brains”. Organoids are structures resembling a whole organ and are generated starting from stem cells that develop and differentiate in three-dimensional systems. In 2013, Lancaster from the group of Knoblich [144, 145], developed a protocol for deriving cerebral organoids from induced pluripotent stem cells (iPSCs), which were first cultured as embryoid bodies and then induced to differentiate towards the neuroectoderm. The cells, embedded in a Matrigel pellet, were cultured in a differentiation medium in the presence of EGF/FGF2 and then moved to a spinning bioreactor. These cerebral organoids developed different and interdependent brain regions such as the cerebral cortex, with progenitor cells that self-organized to originate mature cortical neurones. In 2016, Hubert et al. [110] applied this “mini-brain” model to glioblastoma in order to generate brain tumour organoids. These organoids grew for months and showed regional heterogeneity, with an outer region of quickly dividing cells that were positive for stemness marker such as SOX2, OLIG2, and TLX. Importantly, cells of this region were sensitive to radiotherapy, while senescent and quiescent cells of the hypoxic core were overall more radiation resistant. Furthermore, a hypoxic gradient that spatially correlated with reduced SOX2 expression could be observed within the organoid. Successful orthotopic implantation into mouse brains demonstrated the tumorigenic capacity of this model. This work shows that brain tumour organoids can be employed to study tumour heterogeneity and drug sensitivity in a sophisticated model of the primary patient tumour, with a superior level of complexity compared to simple patient-derived sphere cultures.

Recently, a biobank of patient-derived glioblastoma organoids, recapitulating the mutational profiles of the parental tumours, has been generated [146]. This biobank can be interrogated to test personalised therapies by correlating the glioblastoma organoid mutational profiles with drug responses. Overall, compared to conventional cell cultures, tumour organoids better recapitulate the original tumour architecture and its microenvironmental gradients. Additionally, they preserve the cellular heterogeneity of the parental tumour and, for that, they represent a valid option for pre-clinical research on glioblastoma.

### 3D bioprinted GBM models

Due to the process of self-assembly, tumour organoids may have a variable cellular composition and structure. In order to gain a better control over both the cellular and the ECM components, 3D bio-printed models of glioblastoma tumours have been developed. This technology exploits novel biomaterials and recent advances of tissue engineering techniques to reconstruct 3D models that are based on clinical images of the tumour. These images are sliced into 2D sections such that a bioprinter can generate well-defined structures in all three dimensions. A variety of natural or synthetic biocompatible scaffolds that can reproduce the brain ECM are available, like hydrogels of chitosan-alginate and hyaluronic acid, or synthetic polymers like poly-lactide co-glycolide and polyethylene-glycol [147–149]. The bio-printing can be performed using a so-called extrusion method, which consists of the continuous depositing, layer-by-layer, of filaments of biomaterial or of droplets released from a nozzle. Alternatively, photo-crosslinking can be used to induce the photopolymerization of bioink to form 3D structures [150]. Several cell types can be encapsulated into this bio-printed ECM, such that a “mini-brain” structure, replicating the tumour architecture and the interactions among different cell types, can be built. The choice of cellular components, biomaterial and bio-printing method depends on the biological question to be addressed. For example, Heinrich and colleagues developed a brain model in which they first printed a scaffold that encapsulated mouse macrophages. These were placed such that a cavity was left empty for being filled with methacryloyl/gelatin bioink-embedded mouse glioblastoma cells. Finally, the whole construct was photo-crosslinked [111]. In this 3D bio-printed mini-brain the location of the tumour allowed cellular cross-talk to occur similar to that in an *in* *vivo* situation and, therefore, the model was used to study interactions between cancer cells and macrophages. Hermida and colleagues [151] instead used the extrusion method to establish a model comprising glioblastoma cells and stromal cells co-printed in a matrix of alginate, hyaluronic acid and collagen-1 crosslinked with calcium. This also opens the door to structure and define the immune components of GBM in the future, an overall highly interesting possibility in the light of the immune suppressive tumour microenvironment in this disease. Although cost-intensive and limited by the availability of suitable biomaterials that do not affect the normal tissue development, 3D-bioprinted models have the advantage of facilitating the study of the tumour microenvironment and its influence on the tumour cells.

## Conclusions and outlook

Overall, glioblastoma remains a major challenge in cancer research and treatment. While conventional cell line models have proven important for initial drug screening purposes and on-target efficacy studies, more advanced experimental models begin to narrow the gap between *in* *vitro* and *in* *vivo* conditions. As described above, each model comes with limitations and advantages in terms of experimental versatility, cost, complexity and reproducibility (Fig. 1B). The choice of the experimental system therefore should be guided by the scientific question to be addressed. For validation and confirmation of robustness of key findings, it is likewise advisable to make use of more than one model system and/or method.

The future will surely provide us with even more advanced solutions and a better understanding for which additional external factors will need to be controlled better. For example, the majority of experimental studies are currently conducted at atmospheric partial oxygen pressures, which substantially exceeds *in* *vivo* conditions and may influence cellular stress responses and experimental outcomes [152]. Similarly, continued efforts are spent on refining and optimising the composition of culture media. Another major hurdle to overcome will be the development of reliable co-culture systems [153], since in these the interplay of GBM cells and immune cells can be studied more reliably. This topic is likewise of central importance for *in* *vivo* studies. Obviously, the immune component is missing in all conventional xenograft settings, mouse humanization strategies are still at an early stage and it may be difficult to extrapolate syngeneic mouse models to human disease settings [154].

The area of model development will surely progress further, and it will therefore be crucial that improvements and further developments will be met with openness, so that optimal experimental designs and more reliable data can advance the field of GBM research.

## Data Availability

Not applicable.
